# Esophageal perforation following bite of inner tube of automobile tyre: An unusual cause of barotrauma

**DOI:** 10.4103/0971-9261.69142

**Published:** 2010

**Authors:** Y. S. Kadian, S. Agarwal, K. N. Rattan, Nityasha

**Affiliations:** Department of Pediatric Surgery, Pt. BD Sharma PGIMS Rohtak, Haryana – 124 001, India; 1 Department of Radiology, Pt. BD Sharma PGIMS Rohtak, Haryana – 124 001, India; 2 Department of Surgery, Pt. BD Sharma PGIMS Rohtak, Haryana – 124 001, India

**Keywords:** Automobile tyre bite, esophagus, perforation

## Abstract

An 8-year-old child presented with severe chest pain and respiratory distress after he accidentally bit an automobile tyre tube which burst into his mouth. The chest radiograph revealed left-sided pneumothorax. Both esophagogram and computed tomogram (CT) scan revealed a perforation of the distal third of esophagus with extravasation of contrast on left side. The patient was treated conservatively with gastrostomy feeds and antibiotics for 5 weeks with a good response.

## INTRODUCTION

The most important cause of barotrauma of chest is mechanical ventilation. Other causes described in the literature are scuba diving, air bag deployment, and inhalational drug abuse.[1] Etiologic factors of barotrauma, especially to the aerodigestive tract have been reported as tractor tyre,[2] fire extinguisher,[3] exploding bottle,[4] and bicycle inner tube.[1] A search of the literature revealed only one other case of esophageal rupture owing to automobile tyre biting in a child reported apart from our case.

## CASE REPORT

**Figure 1:**
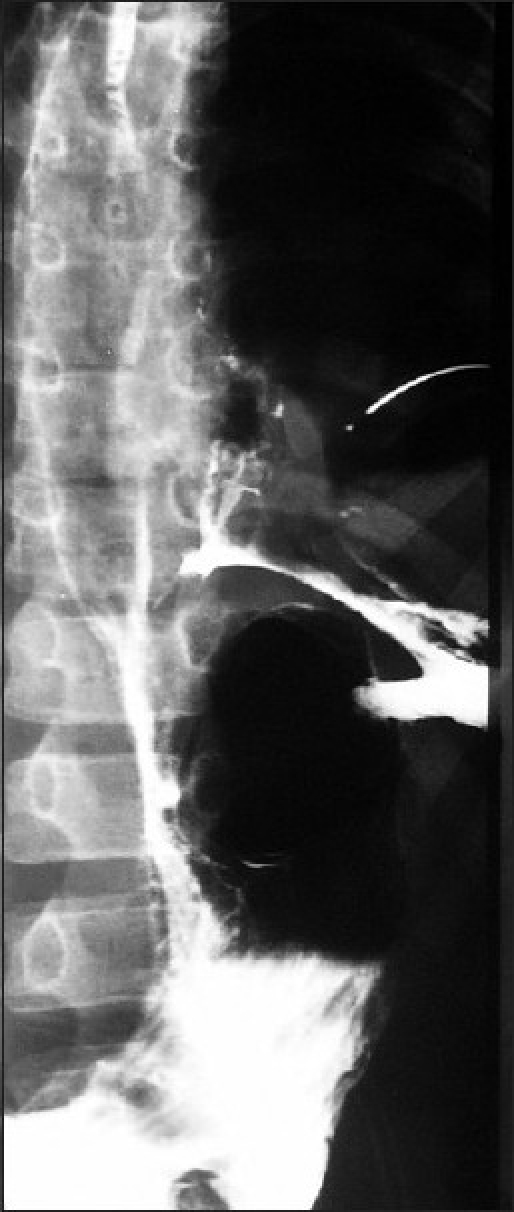
Esophagogram anteroposterior view reveals extravasation of contrast from the distal third of esophagus on left side

An 8-year-old child presented with severe chest pain and respiratory distress after he accidentally bit an agricultural tractor tyre tube, which burst into his mouth. On examination, he had surgical emphysema on left side of the chest extending into neck, and there was decreased air entry on the left side. Radiography of chest revealed left-sided pneumothorax, which was drained by the left intercostal tube. The child was put on ventilator for 48 h, intravenous fluids, and antibiotics. When he was given oral feeds, saliva and milk started coming from left chest drain. Computed tomogram (CT) scan chest and barium swallow [Figure 1] showed a linear tear in the lower third of the esophagus. A feeding gastrostomy was performed and the child was kept nil per oral for 1 month. A repeat barium swallow done after 5 weeks did not show any leak from the esophagus. The child was then started on oral feeds; gastrostomy tube was removed and was discharged in a good condition.

## DISCUSSION

Esophagus is particularly vulnerable at its proximal and distal end because of its anatomy. When there is a sudden and rapid rise in air pressure in the mouth, the pressure is transmitted to the laryngopharynx and enters the respiratory passages, causing a reflex closure of the glottis. As the cricopharyngeus gives way under air pressure, it gives rise to a sudden distention of the esophagus. The cardia fails to relax as it does in the slower, coordinated, reflex act of swallowing, and the dilated esophagus ruptures.[4]

The symptoms of esophageal injuries are often nonspecific and include severe chest pain, dysphagia, or odynophagia.[5] Diagnostic difficulties of esophageal perforations have been emphasized again and again. Both CT scan and esophagogastroscopy have been shown to have limitations. Chien *et al* concluded that optimal tool for the diagnosis of the esophageal perforation is the fluoroscopic esophagography.[6]

Complications of transmural pharyngeal and esophageal injuries include deep neck abscess, mediastinitis, pleural empyema, lung abscess, peritonitis, and sepsis, which are associated with high mortality and morbidity.[4] Brinster *et al*, in a review article, reported that cervical esophageal perforations were associated with a mortality of 6% (0–16%), whereas thoracic and abdominal perforations were associated with mortality of 27% (0–44%) and 21% of patients (0–43%), respectively.[7]

The cases reported in the literature have been managed both surgically and conservatively. Surgical repair is difficult in case of delayed diagnosis, which is associated with high mortality rate (16%–75%) and morbidity rate (35%–66%).[6]

The case described by Özgüner *et al* was a 4-year-old boy who presented with severe dyspnea, subcutaneous emphysema, right-sided pneumothorax, pneumomediastinum, and bilateral emphysema. The diagnosis was made by rigid esophagoscopy, which revealed that there was cervical esophageal rupture. Thereafter he was managed conservatively with gastrostomy feeding for 3 weeks. Oral feeding was started 1 month after the injury.[1] In our patient, there was left-sided pneumothorax and the tear was located in the distal third of esophagus, which was very well delineated with CT scan and esophagogram. Our patient was also conservatively managed with gastrostomy feeding for 1 month. There was no associated orbital emphysema, which was probably due to the location of the tear in the distal esophagus. We are presenting this case to highlight an unusual cause of barotraumatic rupture of esophagus.
